# Mortality for Critical Congenital Heart Diseases and Associated Risk Factors in Newborns. A Cohort Study

**DOI:** 10.5935/abc.20180203

**Published:** 2018-11

**Authors:** Andressa Mussi Soares

**Affiliations:** 1 Hospital Evangélico de Cachoeiro de Itapemirim, Cachoeiro de Itapemirim, ES - Brasil; 2 Faculdade de Medicina da Universidade de São Paulo, São Paulo, SP - Brasil; 3 Departamento de Cardiopatia Congênita e Cardiologia Pediátrica - Gestão 2018-19, RJ - Brasil

**Keywords:** Heart Defects, Congenital/physiopathology, Heart Defects, Congenital/surgery, Infant,Newborn, Mortality, Hypoplastic Left Heart Syndrome, Health Programs and Plans, Maternal and Child Health

Congenital heart disease (CHD) is any change in the anatomy of the heart and its blood vessels that occurs within the first 8 weeks of gestation. The manifestation of CHD is very variable and may occur soon after birth or appear later in childhood or adolescence.

The incidence of CHD is 8 to 10 per 1000 live births, or one in one hundred births. In Brazil, 28,900 children are born with CHD per year (1% of the total birth), of which about 80% (23,800) need cardiac surgery, and half of them need to be operated in the first year of life.^1^

Congenital malformations represent the second main cause of mortality in children under one year of age. CHD is the most frequent and with high mortality in the first year of life in Brazil, and the third cause of death up to 30 days of life.^2^

About five decades ago, nearly 70 percent of children with CHD had an unfavorable outcome and were unable to reach adulthood, as surgery and interventional procedures were not yet available. This panorama has changed much, especially in the developed countries, which have been organized in relation to care in all its stages, from the fetal life to the adult with CHD. In these countries, the life expectancy of newborns (NB) with CHD reaches 85%.^3^^,^^4^

The current national panorama requires urgent measures to improve survival, especially in the neonatal age group. The article "Mortality for Critical Congenital Heart Diseases and Associated Risk Factors in Newborns. A Cohort Study" depicts clearly in a sample of 52 cases of critical CHD, the overall situation of our country, even considering the regional differences. It is known that comprehensive care for the child with CHD in Brazil is still one of the major challenges of Health Unic System (SUS). The continental dimensions of country and the unequal geographical distribution of reference centers of cardiology and pediatric surgery are determining factors in this process.

In this study,^5^ the authors identified that the risk of death in NB infants with CHD was twice as high among premature infants with low birth weight and Apgar < 7 in the first minute of life. The presence of some comorbidity, besides CHD, was associated with the outcome and increased the risk by almost three times. All NB with CHD were placed in the regulation process and did not perform any interventional procedures until the transfer, since none of the maternity hospitals had cardiac surgery services. This reality is frequent in our country, since there are only 69 centers in pediatric cardiac surgery. The average time of hospital stay in this study was 75 days and 25% of the NB with CHD had already died in the neonatal period. The incidence of death in cases of CHD was alarming in a total of 81/100 thousand live births, with cardiogenic shock being the main cause in 41.1% of the cases. Countries in socioeconomic conditions similar to those in Brazil have a global incidence rate of deaths due to CHD of 20 to 30/100 thousand births.^6^

The time of referencing of the NB with critical CHD is proportionally related to mortality, the longer the delay, the higher the mortality, as demonstrated in the study by Fixler et al.,^3^ reaching the next 80% for hypoplastic left heart syndrome.

In 2017, the Brazilian Ministry of Health launched a federal project to expand childcare with CHD,^2^ with the goal of increasing the care of children with CHD per year by 30%, which corresponds to more than 3,400 procedures per year, totalizing about 12,600 procedures / year, which would impact in great reduction of neonatal mortality. The study in question corroborates that CHD care in our country needs intervention, remodeling and restructuring in several phases of its process,^7^ in order to achieve effective goals of reducing the morbidity and mortality of NB and children.

Establishing sustainable cardiac surgery and hemodynamic programs requires more than a financial investment; it involves specific political, social, and cultural issues in each region. Organizations wishing to assist in the development of congenital and pediatric cardiac centers need to focus on two-way communication and education and to maintain a long-term commitment to each location.^8^^,^^9^ The commitment of the nation in several spheres is fundamental to change this panorama in the public health and is a matter of social security in our country.
